# Protracted immune disorders at one year after ICU discharge in patients with septic shock

**DOI:** 10.1186/s13054-017-1934-4

**Published:** 2018-02-21

**Authors:** Florence Riché, Benjamin G. Chousterman, Patrice Valleur, Alexandre Mebazaa, Jean-Marie Launay, Etienne Gayat

**Affiliations:** 1 Department of Anesthesiology and Intensive Care Medicine, Saint Louis Lariboisière University Hospital, University Paris Diderot, Assistance Publique – Hôpitaux de Paris, 2 rue Ambroise Paré, 75010 Paris, France; 2 Inserm U1160, Hôpital Saint-Louis, 1 rue Claude Vellefaux, 75010 Paris, France; 3 Department of Visceral Surgery, Saint Louis Lariboisière University Hospital, University Paris Diderot, Assistance Publique – Hôpitaux de Paris, Paris, France; 4 Biomarkers in CArdio-Neuro-VAScular diseases (BIOCANVAS), UMR-S 942, Inserm, Paris, France; 5 Department of Biochemistry, Saint Louis Lariboisière University Hospital, University Paris Diderot, Assistance Publique – Hôpitaux de Paris, Paris, France

**Keywords:** Sepsis, Late mortality, Protracted immune disorders

## Abstract

**Background:**

Sepsis is a leading cause of mortality and critical illness worldwide and is associated with an increased mortality rate in the months following hospital discharge. The occurrence of persistent or new organ dysfunction(s) after septic shock raises questions about the mechanisms involved in the post-sepsis status. The present study aimed to explore the immune profiles of patients one year after being discharged from the intensive care unit (ICU) following treatment for abdominal septic shock.

**Methods:**

We conducted a prospective, single-center, observational study in the surgical ICU of a university hospital. Eighty-six consecutive patients admitted for septic shock of abdominal origin were included in this study. Fifteen different plasma biomarkers were measured at ICU admission, at ICU discharge and at one year after ICU discharge. Three different clusters of biomarkers were distinguished according to their functions, namely: (1) inflammatory response, (2) cell damage and apoptosis, (3) immunosuppression and resolution of inflammation. The primary objective was to characterize variations in the immune status of septic shock patients admitted to ICU up to one year after ICU discharge. The secondary objective was to evaluate the relationship between these biomarker variations and patient outcomes.

**Results:**

At the onset of septic shock, we observed a cohesive pro-inflammatory profile and low levels of inflammation resolution markers. At ICU discharge, the immune status demonstrated decreased but persistent inflammation and increased immunosuppression, with elevated programmed cell death protein-1 (PD-1) levels, and a counterbalanced resolution process, with elevated levels of interleukin-10 (IL-10), resolvin D5 (RvD5), and IL-7. One year after hospital discharge, homeostasis was not completely restored with several markers of inflammation remaining elevated. Remarkably, IL-7 was persistently elevated, with levels comparable to those observed after ICU discharge, and PD-1, while lower, remained in the elevated abnormal range.

**Conclusions:**

In this study, protracted immune disturbances were observed one year after ICU discharge. The study results suggested the presence of long-lasting immune illness disorders following a long-term septic insult, indicating the need for long-term patient follow up after ICU discharge and questioning the use of immune intervention to restore immune homeostasis after abdominal septic shock.

**Electronic supplementary material:**

The online version of this article (doi:10.1186/s13054-017-1934-4) contains supplementary material, which is available to authorized users.

## Background

Sepsis is a leading cause of mortality and critical illness worldwide [1]. Recent reports have shown that patient mortality has been reduced, particularly when recommended bundles of care are applied [2, 3]. Nevertheless, the septic shock mortality rate still varies between 30 and 40% [2, 3]. In accordance with recent findings, there is growing interest in long-term sepsis outcomes. Sepsis is associated with an increased mortality rate in the months and years after hospital discharge [4]. Observational studies have reported 1-year mortality rates greater than 50% after hospital discharge [5]. The consequences of sepsis, therefore, are not limited to those observed during ICU stays. Post sepsis, patients are prone to decompensating or experiencing several diseases or organ dysfunction, such as cardiovascular disease [6–8], pulmonary disease [9], renal disease [10], depression and cognitive disabilities [11], and immune dysfunction [12]. These ailments lead to long-term decreased functional status [13]. The occurrence of persistent or new organ(s) dysfunction(s) after septic shock raises questions about the cellular, metabolic and immune mechanisms involved in the post-sepsis status.

Potential mechanisms that alter long-term outcomes, such as persistence of altered innate immune and pro-coagulant responses to sepsis, have been previously described. These mechanisms have been studied through several mediators or molecules, such as C-reactive protein (CRP), tumor necrosis factor (TNFα), interleukin (IL)-6, IL-10, d-dimers, antithrombin-III, and factor IX, and the fibrinolytic pathway [12, 14–16]. Another mechanism is the altered adaptive immune response, with depletion of naïve immune cells [17]. Beside usual mediators like inflammatory cytokines, lipid mediators, and protein from the apoptosis cell death machinery, there is growing interest in metabolites involved in inflammation. Tryptophan (Trp) and its metabolites play an important role in the immune balance between response to pathogen and tolerance [18].

The underlying mechanisms explaining the impact of the ICU stay on post-ICU outcomes remain unclear. Accordingly, the present study aimed to explore the natural evolution of the immune profile from ICU admission to 1 year later and to assess the relationship between this immune profile and 1e-year outcomes in ICU patients admitted with abdominal septic shock.

## Method

### Study design

We conducted a single-center, prospective study in a surgical ICU at Lariboisière Hospital, a university hospital located in Paris, France. The main objective was to characterize the global variations in the immune and inflammatory status of patients with septic shock admitted to the ICU, for up to 1 year after hospital discharge, by studying the variations in their biomarkers. The secondary objective was to evaluate the prognostic impact of these biomarkers on patient outcomes. The protocol was approved by our institutional review board (IRB) (Comité d’Evaluation de l’Ethique des Projets de Recherche Biomédicale Paris Nord, IRB 00006477). All included patients were informed about the study, and written informed consent was obtained from all participants. If the patient was unable to be informed, the next of kin was informed and provided consent for the patient to participate.

### Patient selection and data collection

All consecutive patients admitted to the ICU with a diagnosis of septic shock of abdominal origin from February 2010 to May 2013 were enrolled. In all cases, the source was controlled by surgery. Septic shock was defined according to the criteria of the International Sepsis Definitions Conference [19]. Patients younger than 18 years of age, pregnant patients, and patients with aplasia or immunosuppressive disease (e.g., HIV) or receiving immunosuppressive therapy (i.e., chemotherapy, chronic used of steroids, autoimmune disease treatment) were excluded from the study. None of the patients received steroids during their ICU stay. Note that these patients were included in another previously published study (86 of 130 patients) [20]. The following demographic and clinical data were collected: age, sex, comorbidities (Charlson comorbidity index score) [21], Simplified Acute Physiology Score II [22], Sequential Organ Failure Assessment score [23] at day 1, length of ICU stay, ICU mortality, post-ICU discharge mortality, and 1-year survival. We extracted marker measurements in 32 patients post orthopedic surgery, as a control group.

### Studied biomarkers

We studied 15 different plasma biomarkers measured at ICU admission, at ICU discharge in surviving patients, and at 1 year, and grouped within three different clusters. The first cluster was composed of inflammatory cytokines: TNFα, IL-6, interferon-γ (INF-γ), IL-17 and metabolites: L-kynurenine (Kyn), Trp, and indoleamine 2, 3 dioxygenase (IDO). The second cluster was composed of cell damage and apoptosis markers: uric acid, high mobility group Box 1 protein (HMGB1) and caspase-3 activity. Caspase-3 activity was chosen as a surrogate for pro-apoptotic balance. The third cluster was composed of markers of (1) immunosuppression with the programmed death-1 (PD-1) protein and (2) resolution of inflammation with two cytokines - IL-10 and IL-7 - and two plasma lipids that are pro-resolving mediators, resolvins 1 and 5 (RvD1 and RvD5) (Additional file 1: Table S1).

### Laboratory procedures

Data collection was performed within (1) 24 h of the onset of shock, (2) on the day of ICU discharge, and (3) during an ambulatory care visit 12 to 18 months after ICU discharge.

The biomarker analyses were blinded. The plasma levels of IL-6, IL-7, IL-10, IL-17, IFN-γ and TNFα were measured using sandwich immunoassay methods with commercially available electrochemiluminescent detection systems, plates and reagents (V-PLEX human cytokine 30-Plex kits (Meso-Scale Discovery (MSD), Gaithersburg, USA), as per the manufacturer’s instructions. Briefly, 50 μL of plasma was loaded per well in the MSD plates. The plates were analyzed using the SECTOR Imager 2400. Plasma Trp and Kyn levels were measured using high-performance liquid chromatography, as described by Kema et al. [24] and Fujigaki et al. [25], respectively. IDO (*EC* 1.13.11.52) activity was estimated with the Kyn/Trp ratio. Plasma lipid pro-resolving mediators (RvD1 and RvD5) were investigated using liquid chromatography-tandem mass spectrometry (LC-MS-MS), as described by Colas et al. [26]. Caspase-3 activity was determined using the substrate DEVD-AFC in the presence or absence of the caspase-3 inhibitor Ac-DEVD-CHO (Calbiochem), as described by the manufacturer (Abcam). The caspase-3 activity was calculated by subtracting the activity in the presence of Ac-DEVD-CHO from the activity in its absence. The concentrations of circulating high mobility group box 1 (HMGB1) and soluble PD-1 (sPD-1) proteins were measured in plasma samples using commercially available enzyme-linked immunosorbent assay (ELISA) kits (Shino-Test Corporation, Tokyo, Japan and R&D Systems, Minneapolis, MN, USA), according to the manufacturers’ instructions. Finally, plasma uric acid was quantified by colorimetry (uricase assay) on an Architect C8000 clinical chemistry device (Abbott). For each assay, the samples were analyzed in triplicate and then compared with the known concentrations of protein standards, either external or added to the samples to account for any interfering product.

### Statistical analysis

The results are expressed as the median (interquartile range (IQR)) or count (percentage), as appropriate. Two outcome measures were considered: in-ICU mortality in all patients and 1-year mortality in the in-ICU survivors. Comparisons were performed using the Wilcoxon signed-rank test or the chi-squared test, as appropriate. Correlation was assessed using Pearson’s correlation coefficient. A *p* value <0.05 was considered to be statistically significant. All statistical analyses were performed using the R statistical software (The “R” Foundation for Statistical Computing, Vienna, Austria).

## Results

### Patients and outcome

Eighty-six patients with septic shock were enrolled in our study. Thirty-one patients (36%) died in the ICU. Of the 55 ICU survivors, 9 (16%) died in the following year. Table 1 shows the baseline characteristics of the cohort. The origins of sepsis were primarily peritonitis, biliary diseases, and acute intestinal ischemia.

**Table 1:** Patients’ characteristics

	All patients (*n* = 86)
Age, years	72 (62–81)
Female gender	41 (48%)
Origin of abdominal sepsis	
Community-acquired/postoperative peritonitis	26 (30%)/23 (27%)
Acute biliary diseases: angiocholitis, biliary peritonitis	14 (16%)
Acute intestinal ischemia	20 (23%)
Miscellaneous	3 (3%)
Comorbidities	
Heart and vascular diseases	23 (30%)
COPD	3 (3%)
Cancer/immunosuppression	19 (22%)
Charlson comorbidity index score	3 (2–4)
Severity and outcome	
SAPS II	50 (44–60)
SOFA score at admission	9 (7–12)
In-ICU LOS (days)	8 (4–17)
In-ICU deaths	31 (36%)
In-hospital LOS (days)	23 (8–47)
Death at 1 year among ICU survivors	9 (16%)

Continuous variables are expressed as median (interquartile range) or count (percentage), as appropriate

*Abbreviations*: *COPD* chronic obstructive pulmonary disease, *SAPS II* Simplified Acute Physiology Score II, *SOFA* Sequential Organ Failure Assessment, *ICU* intensive care unit, *LOS* length of stay

### Biomarker profiles from onset to one year after septic shock

**Fig. 1:**
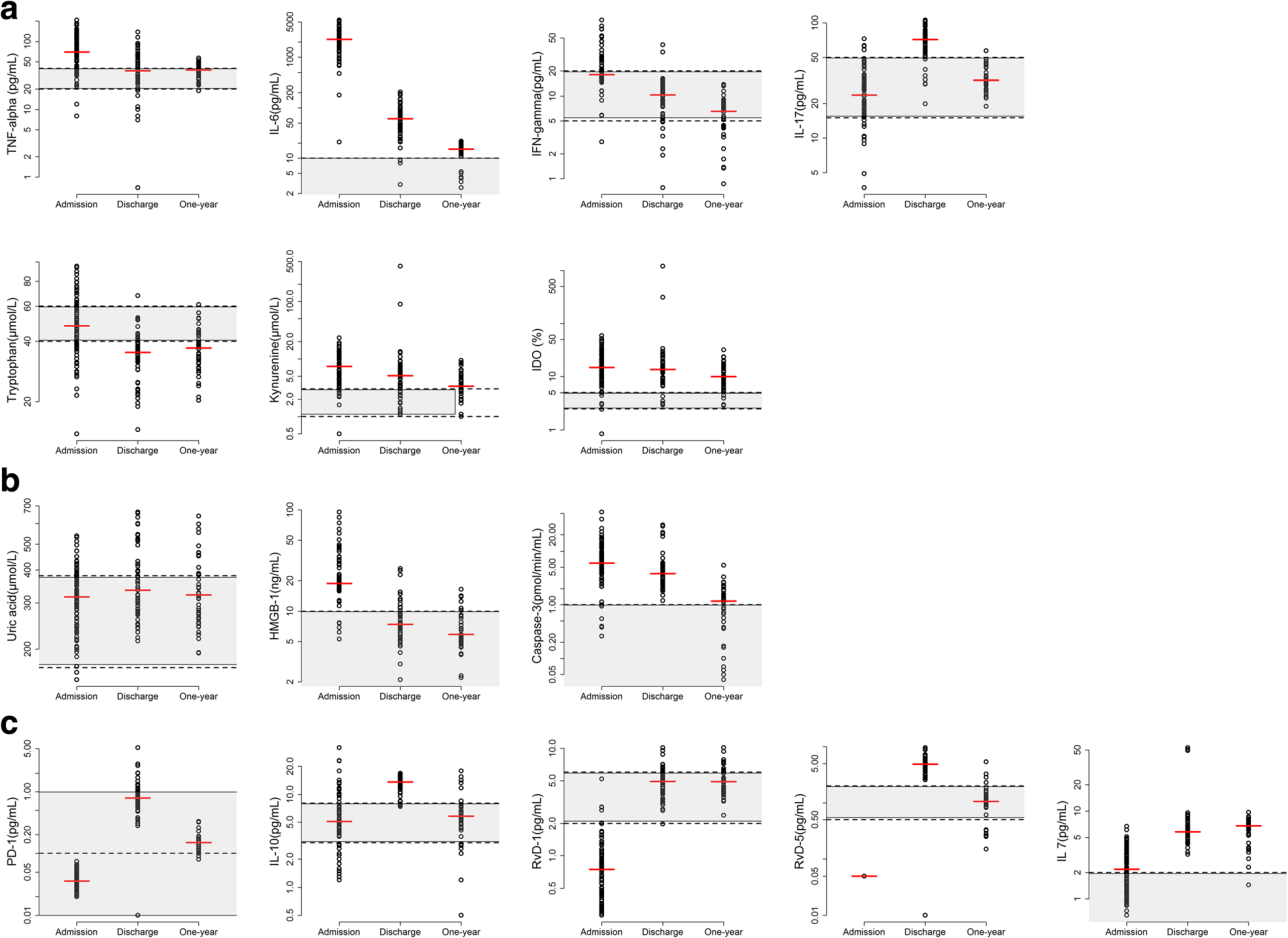
Biomarker profile at the onset of septic shock, at ICU discharge and up to one year following septic shock. **a** biomarkers of inflammation, **b** biomarkers of damage and apoptotic cells, **c** biomarkers of sepsis induced immunosuppression and resolution of inflammation. *TNF* tumor necrosis factor, *IL* interleukin, *IFN-γ*, interferon-γ, *IDO* indoleamine2,3 dioxygenase, *HMGB1* high mobility group box 1 protein, *PD* programmed cell death protein, *RvD* resolvins

Values of biomarkers at admission, at discharge and at one year are depicted in Fig. 1. Table 2 summarizes the corresponding values compared to a control group and reports the proportion of patients outside the normal range for each biomarkers at the three timepoints.

**Table 2:** Biomarkers levels at admission, ICU discharge, and at one year

	Control group (*n* = 32)	Admission (*n* = 86)		Discharge (*n* = 55)		One year (*n* = 46)		*p* value
		Median (IQR)	Proportion outside the normal range^a^	Median (IQR)	Proportion outside the normal range^a^	Median (IQR)	Proportion outside the normal range*	
TNFα (pg/mL)	23.8 (20.8–34.5)	65 (41.2–95.5)	78%	35.2 (21–54)	45%	37.8 (32.2–44.5)	33%	<0.0001
IL-6 (pg/mL)	5.7 (3.5–8.2)	2289 (1487–3050)	99%	59 (30.5–83)	91%	15.2 (13.4–16.8)	78%	<0.0001
IFNɣ (pg/mL)	9.1 (7.4–15.8)	19.2 (17.1–27.1)	34%	10 (7.4–13.8)	4%	6.6 (4.2–8.3)	0%	<0.0001
IL-17 (pg/mL)	19.5 (16.9–27.9)	20.1 (16–28.5)	5%	70.8 (55.9–79.5)	87%	31.8 (29.8–40.9)	2%	<0.0001
Trp (μmol/L)	45.5 (41.9–49.1)	48.9 (39.7–60.8)	74%	35.7 (29.2–40.9)	98%	37 (31–44)	89%	<0.0001
IDO (%)	3.9 (3.4–5.5)	11.1 (8.5–15.6)	87%	13.5 (9.2–18.5)	78%	10.1 (7–15.1)	63%	<0.0001
Kyn (μmol/L)	1.8 (1.5–2.5)	5.6 (3.7–7.9)	94%	5.1 (3.4–6.1)	91%	3.3 (2.8–4.9)	63%	<0.0001
Uric acid (μmol/L)	289 (254.8–326.5)	323 (278.2–381)	30%	318 (274–397.2)	33%	321.5 (262–388)	22%	0.04
HMGB-1 (ng/mL)	6.7 (4–8)	17.3 (16.1–18.9)	94%	7 (5.5–9.3)	24%	5.9 (5.2–8.9)	15%	0.61
Caspase 3 (pmol/min/mL)	0.5 (0.4–0.6)	4.5 (2.2–7.6)	83%	3.8 (2.5–4.9)	100%	1.2 (0.4–1.8)	48%	0.01
PD-1 (ng/mL)	0.05 (0.04–0.10)	0.04 (0.03–0.05)	0%	0.79 (0.56–1.03)	95%	0.15 (0.12–0.18)	80%	<0.0001
IL-10 (pg/mL)	5.3 (3.8–6.5)	6.1 (4.1–7.4)	21%	13.8 (11.6–15.5)	91%	5.8 (4.3–7.3)	15%	0.45
RvD1 (pg/mL)	3.5 (2.8–5.2)	0.8 (0.4–1)	0%	5 (3.9–5.8)	22%	4.9 (4.1–6.1)	26%	0.002
RvD5 (pg/mL)	1.4 (0.6–1.5)	0 (0–0)	0%	4.9 (4–6.1)	95%	1.1 (0.8–1.3)	9%	0.27
IL-7 (pg/mL)	0.6 (0.4–1.3)	2.2 (1.2–3.2)	56%	5.8 (4.8–7.1)	100%	6.8 (5.7–7.4)	89%	<0.0001

Continuous variables are expressed as median (interquartile range); *p* values are from the Wilcoxon test comparing patients at 1 year with the control group

*Abbreviations*: *TNFα* tumor necrosis factor alpha, *IL* interleukin, *IFN* interferon, *Trp* tryptophan, *Kyn* kynurenine, *IDO* indoleamine2,3 dioxygenase, *HMGB* high mobility group box 1 protein, *PD*-1, programmed death protein-1, *RvD* resolvins

^a^Normal ranges were defined according to manufacturers’ specifications as reported in Additional file 1: Table S1

#### Biomarkers at admission

At the onset of septic shock, we observed a cohesive pro-inflammatory profile. The primary findings were augmented levels of TNFα, IL-6, Kyn and IDO for the first cluster and HMGB1 and caspase 3 for the second cluster. Conversely, cluster 3 biomarkers, RvD1 and RvD5, were diminished.

#### Biomarkers at discharge

At the time of ICU discharge, the profile evolved, tending toward the resolution of the inflammation. Nevertheless, except for TNFα and IFN-γ, there were abnormal levels of all biomarkers of the inflammatory cluster. Pro-inflammatory mediators (IL-6, IL-17, Kyn and IDO) were elevated. Remarkably, the levels of IL-6 decreased compared to admission levels, whereas IL-17 increased.. The apoptotic biomarker, caspase-3, had decreased, but it was still abnormally high. Immunosuppression starts with increased PD-1, together with resolution of the inflammation process, as reflected in the elevated levels of IL-10, RvD1, RvD5, and IL-7.

#### Biomarkers at one year

At 1 year, there was no complete restoration of homeostasis. Several markers had persistent abnormal levels, while some had corrected and returned to normal levels. IL-6, Kyn, and IDO in cluster 1, caspase 3 for cluster 2 and PD-1 and IL-7 in cluster 3 were significantly elevated. Note that the PD-1 levels were above the normal range but were lower compared to the ICU discharge levels, while the IL-7 levels were consistent with those observed at discharge. Accordingly, the Trp level was below the normal range. The proportion of patients having at least one abnormal biomarker in only one cluster was 9% (n = 4), whereas 70% (n = 32) had abnormal values of biomarkers within all three clusters.

**Fig. 2:**
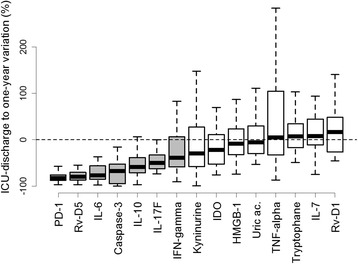
Variation in circulating biomarkers from ICU discharge to one year. *TNF* tumor necrosis factor, *IL* interleukin, *IFN* interferon, *IDO* indoleamine2,3 dioxygenase, *HMGB1* high mobility group box 1 protein, *PD-1* programmed cell death protein-1, *RvD* resolvins

Regarding the difference between the measurements taken at ICU discharge and at 1-year follow-up, we identified two types of biomarkers of change: those associated with a significant decrease (i.e., PD-1, RvD5, IL-6, caspase 3, IL-10, IL-17 and INF-γ) and those associated with no significant change from discharge to 1 year (i.e., Kyn, IDO, HMGB1, uric acid, TNFα, Trp, IL-7, and RvD1) (Fig. 2).

#### Association between biomarkers and prognosis

**Fig. 3:**
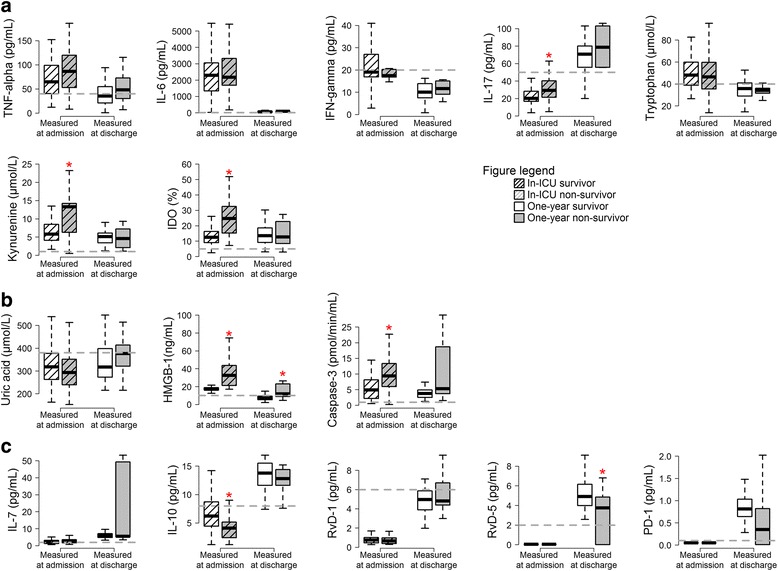
Levels of biomarkers according to patients’ outcomes. Red stars indicate a significant difference in biomarker level between survivors and non-survivors. **a** biomarkers of inflammation, **b** biomarkers of damage and apoptotic cells, **c** biomarkers of sepsis induced immunosuppression and resolution of inflammation. *TNF* tumor necrosis factor, *IFN* interferon, *IDO* indoleamine2,3 dioxygenase, *HMGB1* high mobility group box 1 protein, *RvD* resolvins

The extent of inflammation correlated with ICU outcomes. ICU non-survivors had higher levels of cluster 1 biomarkers (IL-17, Kyn, and IDO activity) and cluster 2 biomarkers (HMGB1 and caspase 3), while their IL-10 levels (cluster 3) were lower at admission (Fig. 3, Table 3). At ICU discharge, no cluster 1 biomarker was associated with 1-year mortality. Interestingly, the cell damage marker (HMGB1) and the resolving RvD5 levels were higher and lower, respectively, at ICU discharge in the 1-year non-survivors (Fig. 3, Table 4). The area under the receiver operating characteristic (ROC) curves for these two biomarkers were 0.743 (0.5–0.937) and 0.715 (0.507–0.903) for HMGB1 and RvD5, respectively. After adjustment for SOFA score at admission, both biomarkers were independently associated with 1-year outcome (odds ratios 1.32 (1.07–1.61) (*p* = 0.009) and 0.58 (0.37–0.90) (*p* = 0.016) for an increase of one unit of HMGB1 and RvD5, respectively).

**Table 3:** Association between biomarkers levels and outcome

	In-ICU survivors *n* = 55	In-ICU non survivors *n* = 31	*p* value
Circulating biomarkers at ICU admission			
TNF alpha (pg/mL)	64.5 (40.4–98.8)	86.2 (52.8–119.8)	0.23
IL-6 (pg/mL)	2286.7 (1342–3013.9)	2167 (1685.5–3317)	0.59
IFN-gamma (pg/mL)	19 (16.9–26.9)	17.4 (16.8–20.2)	0.43
IL-17 (pg/mL)	20.1 (16.7–28.3)	29.3 (21.4–40.2)	0.002
Tryptophan (μmol/L)	48 (38.8–59.8)	46.4 (35.4–59.8)	0.56
IDO (%)	12.5 (9–16.2)	24.7 (15.2–32.5)	<0.0001
Kynurenine (μmol/L)	5.8 (4.1–8.4)	13.3 (6.3–14.2)	<0.0001
Uric acid (μmol/L)	318 (262.5–377)	293 (239.5–351.5)	0.14
HMGB1 (ng/mL)	17 (16.1–18.9)	32.6 (21.1–43.7)	<0.0001
Caspase-3 (pmol/min/mL)	4.9 (2.2–8.1)	9.4 (6–13.3)	<0.0001
PD-1 (ng/mL)	0 (0–0)	0 (0–0)	-
IL-10 (pg/mL)	6.2 (4.5–8.7)	4.1 (2.4–5.2)	0.001
RvD1 (pg/mL)	0.8 (0.5–1)	0.7 (0.4–1)	0.4
RvD5 (pg/mL)	0 (0–0)	0 (0–0)	-
IL-7 (pg/mL)	1.9 (1.3–3.1)	2.6 (1.7–3.6)	0.14

Results are expressed as median (interquartile range); *p* values are from the Wilcoxon rank test

*Abbreviations*: *TNF* tumor necrosis factor, *IL* interleukin, *IFN* interferon, *IDO* indoleamine2,3 dioxygenase, *HMGB* high mobility group box 1 protein, *PD* programmed cell death, *RvD* resolvins

**Table 4:** Association between biomarkers levels and outcome

	One-year survivors *n* = 46	One-year non survivors *n* = 9	*p* value
Circulating biomarkers at ICU discharge			
TNF alpha (pg/mL)	35.2 (21–54)	48 (30–72.7)	0.25
IL-6 (pg/mL)	59 (30.5–83)	86 (51–89.3)	0.26
IFN-gamma (pg/mL)	10 (7.4–13.8)	11.6 (9–15.1)	0.24
IL-17 (pg/mL)	70.8 (55.9–79.5)	78.7 (55.7–103.1)	0.13
Tryptophan (μmol/L)	35.7 (29.2–40.9)	34.1 (31.5–36.1)	0.67
IDO (%)	13.7 (9.2–18.7)	12.7 (8.4–22.7)	0.7
Kynurenine (μmol/L)	5.1 (3.4–6.1)	4.6 (2.1–7.2)	0.55
Uric acid (μmol/L)	317.5 (273.5–397.2)	376 (321–414)	0.35
HMGB1 (ng/mL)	7 (5.5–9.3)	11.8 (8.8–23)	0.023
Caspase-3 (pmol/min/mL)	3.8 (2.5–4.9)	5.3 (3.7–18.7)	0.13
PD-1 (ng/mL)	0.8 (0.6–1)	0.4 (0–0.8)	0.057
IL-10 (pg/mL)	13.8 (11.6–15.5)	12.8 (11.6–14.4)	0.33
Rv-D1 (pg/mL)	5 (3.9–5.8)	4.8 (4.4–6.7)	0.36
Rv-D5 (pg/mL)	4.9 (4–6.1)	3.8 (0–4.8)	0.044
IL-7 (pg/mL)	5.8 (4.8–7.1)	5.5 (5.2–49.3)	0.77

Results are expressed as median (interquartile range); *p* values are from the Wilcoxon rank test

*Abbreviations*: *TNF* tumor necrosis factor, *IL* interleukin, *IFN* interferon, *IDO* indoleamine2,3 dioxygenase, *HMGB* high mobility group box 1 protein, *PD* programmed cell death, *RvD* resolvins

### Clinical signs at baseline and markers levels variations

Correlation with SOFA score at admission and variations in biomarker levels from discharge to 1 year are shown in Additional file 1: Table S2. There was no significant correlation with variation, except for moderate correlation between variation in kynurenine and the SOFA score (rho (95% CI)) = 0.23 (-0.04; - 0.47)).

## Discussion

This study aimed to explore the evolution of the immune profile of patients admitted to the ICU for septic shock of abdominal origin up to 1 year after ICU discharge. Thus, we studied several plasma biomarkers at ICU admission, discharge and 12 months after ICU discharge. The primary result was a protracted immune disturbance 1 year after ICU discharge, Several markers had persistent abnormal levels; IL-6, Trp, Kyn and IDO in cluster 1, caspase 3 in cluster 2, and PD-1 and IL-7 in cluster 3 were significantly elevated. The proportion of patients having at least one abnormal biomarker in only one cluster was 9% (*n* = 4), whereas 70% (*n* = 32) had abnormal values of biomarkers within all three clusters.

The study of the variations from ICU discharge to 1 year identified two types of biomarkers of change: those associated with a significant decrease (i.e., PD-1, RvD5, IL-6, caspase 3, IL-10, IL-17 and INF-γ) and those associated with no significant change from discharge to 1 year (i.e., Kyn, IDO, HMGB1, uric acid, TNFα, Trp, IL-7, and RvD1).

We also explored the relationship between the biomarker levels and patient outcomes. At admission, the patients who died in the ICU had higher levels of inflammatory biomarkers (IL-17, Kyn, IDO) and cell damage biomarkers (HMGB1, caspase 3), while levels of IL-10, an anti-inflammatory cytokine, were lower compared to levels in the survivors. At ICU discharge, HMGB1 was higher and RvD5 was lower in the patients who died in the first year after sepsis compared to the survivors. These results highlight the long-term sequelae of septic insult.

It is now well-recognized that patients with sepsis continue to have the worst outcomes in the months and years after hospital discharge; there is growing interest in the study of long-term sepsis outcomes. Several reports have shown that sepsis survivors are likely to develop chronic illness, such as cardiovascular [8], pulmonary [9], and renal [10] disease, or depression [11]. Our study findings align with those of prior studies. Persistent inflammation, with elevated levels of IL-6 and IL-10, was found at hospital discharge after community-acquired pneumonia [12].

Little is known about the disease mechanistic pathways involved in this phenomenon. To explore this pathogenesis, we conducted a wide study of the immune profile over time. We did not limit our work to inflammation but extended it to the markers of cell damage, apoptosis, immunosuppression and resolution of inflammation, to paint a picture of an evolving mechanism beginning with tissue damage that causes inflammation, followed by a healing process to restore homeostasis.

There is a discrepancy between the clinical picture of “cured” patients who are eligible for ICU discharge (for whom dampening of the inflammation is expected to be rather quick) and evidence of persistent sickness with a damaged immune response that remains long after the event. This discrepancy emphasizes the absence of a homeostasis reset after curing visible signs of sepsis, which could lead to the development or acceleration of chronic diseases.

Most of the studied markers tended to return (more or less) to their normal levels. The clinical significance of moderately elevated IL-6, Kyn, and IDO is questionable. In addition, long-term mild inflammation has been associated with bad outcomes in various diseases, such as cancer [27] or atherosclerosis [28]. Trp and its metabolites play an important role in the immune balance between response to pathogen and tolerance [18]. Trp, an essential amino-acid, is the serotonin precursor and Trp deficits are correlated with impaired T cell proliferation [29] and enhancement of cellular stress response. The intracellular enzyme, IDO degrades Trp and produces Kyn-derived metabolites. IDO is stimulated by lipopolysaccharide (LPS), TNFα, IFN-γ, transforming growth factor (TGF)ß and activation of toll-like receptor (TLR)3, TLR4, TLR7, and TLR8 [30]. After major trauma, increased plasma Kyn and Kyn-Trp ratios are early indicators for sepsis development [31]. Kyn directly damages tissues and organs, induces dysregulation of vascular tone, and is a key factor in the communication between the nervous and immune systems. Kyn metabolites such as kynurenic and quinolinic acids could also impair neurological systems [32].

Trp levels increased from ICU discharge to 1 year, and while this increase is supposed to be beneficial, the levels remained below the control group values at 1 year. Low Trp levels are reportedly associated with cancer [33], cardiovascular disease [34], and depression [35]. Integrative studies using several immuno-metabolic markers and clinical score were found to be useful to predict the risk but could also help in the understanding of the pathophysiology of septic derangements [36, 37]. PD-1, which has an immunosuppressive function as a negative regulator of T cells by preventing their proliferation and altering their bacterial clearance, was still elevated at 1 year, although this biomarker level dramatically decreased (80%) after ICU discharge. Together, these findings raise the hypothesis of a protracted immunodeficiency state.

The elevated levels of IL-7 were surprising. This report is the first to focus on circulating IL-7 at 1 year after septic shock and indicates an ongoing immune process. IL-7 is thought to be a beneficial mediator after acute inflammation. It has an anti-apoptotic effect on lymphocytes and has stimulating effects on B lymphocytes and natural killer cells [38]. These findings raise the question of a lasting immune healing response and may indicate a possible treatment for post-sepsis immune dysfunction, because IL-7 administration has already been proposed for immune depression in HIV infection [39]. The effect of IL-7 on long-term outcomes must be further investigated.

### Limitations of the study

Our cohort was monocentric and relatively small, in terms of the high number of studied biomarkers. It is, therefore, possible that some of our conclusions are erroneous and must be confirmed in larger prospective multicentric studies. We also studied a specific group of patients (i.e., those with septic shock of abdominal origin), which limits the generalizability of our results. The choice of patients with septic shock and not patients with severe sepsis was based on the hypothesis that the sickest patients have worse symptoms and would be more easily identified among a limited number of subjects. We had to choose a limited number of biomarkers. We cannot exclude that other molecules could have been of interest. In addition, we did not study cell function or number at 1 year. Our study was focused on the variation of the biomarkers levels during and after ICU admission and discharge. The association between the biomarkers levels and prognosis was a secondary objective and therefore no sample calculation was made. Conclusions that can be drawn from these data are limited.

Source control may impact the kinetics of the measured mediators. All our patients had the same type of source control (i.e., surgery). Despite the fact that the time and type of surgery could play a role in patient disease severity, we believe that they would marginally impact the levels of mediators at the studied time points. More important, we do not have any data on the relationships between the biomarker levels and outcomes other than death. Such data were, unfortunately, not recorded. The control group was not matched on age, sex, and comorbidities, limiting the interpretation of the results. Another limitation is that we could not assess the levels of biomarkers in our patients before they had septic shock; these could have been abnormal before the onset of sepsis. There is a long gap between ICU discharge and 1 year. The exclusion of nine patients who died during this period may bias the results. If measurements were obtained at 3 and 6 months, more abnormalities may have been detected.

## Conclusion

The present study underscores the notion of a long-lasting, immune illness disorder after septic insult. It also suggests the need to examine the immune and cell damage responses not only at the beginning of ICU admission but also at ICU discharge and much later, up to 1 year.

A better understanding of those disorders might help to delineate groups of patients with different manifestations of acute septic illness. Follow up should be performed to identify specific and appropriate treatments, even long after the acute septic insult. For example, in our study, the persistence of high circulating PD-1 may indicate treatment by anti-PD1. Likewise, the beneficial high levels of circulating IL-7 in the 1-year survivors may indicate the importance of monitoring these levels and considering administration of this beneficial mediator following ICU care. Obviously, further studies are needed to confirm those hypotheses.

## Additional file

Additional file 1: Details on biomarker clusters. **Table S1.** Four clusters of circulating mediators of immune response. **Table S2.** Correlation between biomarkers at 1 year and SOFA score at admission. (DOCX 60 kb)
