# Supplementary material for: Protracted immune disorders at one year after ICU discharge in patients with septic shock
Source: Crit Care. 2018 Feb 21;22:42. doi: 10.1186/s13054-017-1934-4 (PMC5822646; doi:10.1186/s13054-017-1934-4)
Supplement: Supplementary file 1 — Details on biomarker clusters. Table S1. Four clusters of circulating mediators of immune response. Table S2. Correlation between biomarkers at 1 year and SOFA score at admission. (DOCX 60 kb) [file 13054_2017_1934_MOESM1_ESM.docx]

Protracted immune disorders in septic shock patients one year after ICU discharge

Florence Riché, Benjamin G Chousterman, Patrice Valleur, Alexandre Mebazaa, Jean-Marie Launay and Etienne Gayat

**.**

**Additional file**

**Corresponding author:** Dr Florence Riché, MD, PhD

Département d’Anesthésie – Réanimation

Hôpitaux Universitaires Saint Louis – Lariboisière

2 rue Ambroise Paré

75010 Paris

Tel : +33 1 49 95 80 71

Email : florence.riche@aphp.fr

**Details on biomarkers’ cluster**

The first cluster examined (i) inflammatory biomarkers (i.e., TNFα, IL-6, INF ϒ (1) and IL-17, which acts as a potent mediator that increases chemokine production in various tissues (2)) and (ii) key immune response biomarkers (i.e., tryptophan [Trp], kynurenine [Kyn] and indoleamine 2,3 dioxygenase [IDO] activity). Tryptophan, the serotonin precursor, is required for protein synthesis. Withdrawal of this essential amino acid stops this process and, subsequently, favors the growth of pathogens (3). IDO, which is produced by monocytes (antigen-presenting cells), is induced by pro-inflammatory cytokines and/or lipopolysaccharide, which activates Toll-like receptors. This induction of IDO activity reduces TRP availability, activates the cellular stress response pathway and produces tryptophan metabolites along the kynurenine pathway. Those metabolites induce T-cell apoptosis, suppress immune responses and impair microvascular reactivity. (4, 5)

The second cluster includes biomarkers of cell damage and apoptosis. Alarmins (uric acid and HMGB1 in the present study) belong to the damage-associated molecular pattern that is released by damaged or necrotic cells in response to infection or injury, and they activate the immune system (6). Apoptosis was analyzed through caspase 3 activity. It is a frequently activated death protease that causes lymphocytes and neutrophils loss (7). The sepsis-induced expression of PD-1 by macrophages contributes to the inhibition of macrophage functions and negatively regulates T cell responses, contributing to an altered microbial clearance (8).

The third cluster concerns mediators that are involved in immunosuppression on the one hand and inflammation resolution on the other hand. Immunosuppression was studied with the programmed death-1 (PD-1) co-receptor. The sepsis-induced expression of PD-1 by macrophages helps to inhibit macrophage functions and negatively regulates T cell responses, thereby altering microbial clearance (8). Concerning the resolution of inflammation, the anti-inflammatory cytokine IL-10 downregulates a wide range of pro-inflammatory cytokines and activates T cells (9). Resolvins 1 and 5 (RvD1, RvD5) are host-protecting lipids that are formed in the early phase of inflammation. They promote bacterial phagocytosis, augment host recovery from infection (by blocking activation of inflammasome components) and attenuate the production of pro-inflammatory eicosanoids (10). IL- 7, a hematopoietic growth factor that prevents lymphocytes apoptosis and stimulates lymphoid lineage cells (**Table S1**)

**Table S1.** Four clusters of immune response circulating mediators

|  | Biomarker | Main Activity | Normal ranges |
| --- | --- | --- | --- |
| **Inflammatory response** |  |  |  |
|  | Tumor necrosis factor(TNFα) | Pro-inflammatory activity of innate immune response (12) | 20-40 pg/ml |
|  | Interleukin-6 (IL-6) | Pro-inflammatory activity of innate immune response ; induces hepatic acute phase proteins. (12) | <10 pg/ml |
|  | Interferon ϒ (INFɣ) | Enhances microbicidal activity of macrophages, up-regulates expression of MHC Class I ,II. (13) | 5-20 pg/ml |
|  | Interleukin-17 ( IL-17) | Increases chemokine production in tissues to recruit monocytes, neutrophils to the site of inflammation, emergency granulopoiesis (2) | 15-50 pg/ml |
|  | Tryptophan  Indoleamine2,3 dioxygenase (IDO)  Kynurenines | Essential amino acid, builds proteins, key immune mediator (3)  Induces by pro-inflammatory cytokines, activates cellular stress response, produce kynurenines, attenuate host T cell response (3, 5)  Suppress immune response , induces T-cell apoptosis , impair vascular reactivity, major player in depressive disorders (4, 14) | 40-60 µmol/L  2,5-5 %  1-3 µmol/L |
| **Damage and apoptotic cells** |  |  |  |
| Damage | Uric Acid | Principal endogenous danger signal. It alerts the immune system to dying cells (15) | F : 140-340 µmol/L  H : 200-420 µmol/L |
|  | High Mobility Group Box 1 protein (HMGB1) | Protein released by necrotic or damaged cells. (6) | <10 ng/ml |
| Apoptosis | Caspase 3 | Proteases , central component of apoptosis cell death machinery, causes lymphocytes, neutrophils lost (7) | <1 pmol/min/ml |
| **Sepsis induced immunosuppression**  **and resolution of inflammation** |  |  |  |
| Immunosuppression | Program Death 1 (PD-1) | Protein expressed on [T , B , NKT cells](https://en.wikipedia.org/wiki/T_cell) and monocytes. PD-1 negatively regulates T cell responses, prevents their proliferation , alters bacterial clearance (8) | <0.1 ng/ml |
| Resolution of inflammation | Interleukin-10 (IL-10) | Anti-inflammatory , down regulates pro-inflammatory cytokines, activate Ly T (9) | 3-8 pg/ml |
|  | Resolvins : RvD1  RvD5 | Lipids mediators, participate in the resolution of inflammation, enhance phagocytic clearance, modulate neutrophil chemotaxis (10) | 2 – 6 pg/ml  0.5- 2 pg/ml |
|  | Interleukin-7 (IL-7) | Prevents Ly T apoptosis.  Stimulates proliferation Ly B, T NK cells. (11) | <2pg/ml |

**Table S2.** Correlation between biomarkers at one-year and SOFA score at admission

| Variation from discharge to one-year (%) | Correlation coefficient with SOFA score at admission [95% CI] |
| --- | --- |
| **TNFalpha** | 0.19 [-0.09 - 0.43] |
| **IL6** | -0.05 [-0.31 - 0.22] |
| **IFNgamma** | 0.11 [-0.16 - 0.37] |
| **IL17F** | 0.06 [-0.21 - 0.32] |
| **Trp** | 0.01 [-0.25 - 0.28] |
| **Kyn** | 0.23 [-0.04 - 0.47] |
| **IDO** | 0.06 [-0.21 - 0.33] |
| **HMGB1** | 0.04 [-0.23 - 0.3] |
| **AU** | -0.12 [-0.37 - 0.16] |
| **caspase** | -0.07 [-0.33 - 0.2] |
| **PD1** | 0.07 [-0.21 - 0.33] |
| **RvD1** | 0.15 [-0.12 - 0.41] |
| **RvD5** | 0.1 [-0.17 - 0.36] |
| **IL7** | -0.12 [-0.37 - 0.16] |
| **IL10** | -0.18 [-0.42 - 0.1] |
| **ratio** | -0.10 [-0.35 - 0.18] |

**References**

1. Cohen J. The immunopathogenesis of sepsis. Nature. 2002;420(6917):885-91.

2. Miossec P, Korn T, Kuchroo VK. Interleukin-17 and type 17 helper T cells. N Engl J Med. 2009;361(9):888-98.

3. Mellor AL, Munn DH. IDO expression by dendritic cells: tolerance and tryptophan catabolism. Nat Rev Immunol. 2004;4(10):762-74.

4. Darcy CJ, Davis JS, Woodberry T, McNeil YR, Stephens DP, Yeo TW, et al. An observational cohort study of the kynurenine to tryptophan ratio in sepsis: association with impaired immune and microvascular function. PLoS One. 2011;6(6):e21185.

5. Frumento G, Rotondo R, Tonetti M, Damonte G, Benatti U, Ferrara GB. Tryptophan-derived catabolites are responsible for inhibition of T and natural killer cell proliferation induced by indoleamine 2,3-dioxygenase. J Exp Med. 2002;196(4):459-68.

6. Chan JK, Roth J, Oppenheim JJ, Tracey KJ, Vogl T, Feldmann M, et al. Alarmins: awaiting a clinical response. J Clin Invest. 2012;122(8):2711-9.

7. Porter AG, Janicke RU. Emerging roles of caspase-3 in apoptosis. Cell Death Differ. 1999;6(2):99-104.

8. Huang X, Venet F, Wang YL, Lepape A, Yuan Z, Chen Y, et al. PD-1 expression by macrophages plays a pathologic role in altering microbial clearance and the innate inflammatory response to sepsis. Proc Natl Acad Sci U S A. 2009;106(15):6303-8.

9. Fiorentino DF, Zlotnik A, Mosmann TR, Howard M, O'Garra A. IL-10 inhibits cytokine production by activated macrophages. J Immunol. 1991;147(11):3815-22.

10. Lee CR, Zeldin DC. Resolvin Infectious Inflammation by Targeting the Host Response. N Engl J Med. 2015;373(22):2183-5.

11. Fry TJ, Mackall CL. Interleukin-7: from bench to clinic. Blood. 2002;99(11):3892-904.

12. Dinarello CA. Historical insights into cytokines. Eur J Immunol. 2007;37 Suppl 1:S34-45.

13. Schroder K, Hertzog PJ, Ravasi T, Hume DA. Interferon-gamma: an overview of signals, mechanisms and functions. J Leukoc Biol. 2004;75(2):163-89.

14. Schwarcz R, Bruno JP, Muchowski PJ, Wu HQ. Kynurenines in the mammalian brain: when physiology meets pathology. Nat Rev Neurosci. 2012;13(7):465-77.

15. Shi Y, Evans JE, Rock KL. Molecular identification of a danger signal that alerts the immune system to dying cells. Nature. 2003;425(6957):516-21.
